# The effects of participating in nursing student congress and other motivation sources on occupational motivation states of Nursing students: Toros University example

**DOI:** 10.12669/pjms.35.1.403

**Published:** 2019

**Authors:** Behire Sancar, Nazife Akan

**Affiliations:** 1 Dr. Behire Sancar, RN, Msc, PhD. Assistant Professor, Department of Nursing and Health Services, Toros University School of Health Science, Mersin, Turkey; 2 Dr. Nazife Akan, RN, Msc, PhD. Assistant Professor, Department of Nursing and Health Services, Toros University School of Health Science, Mersin, Turkey

**Keywords:** Nursing, Nursing student, Motivation, Occupational motivation

## Abstract

**Objective:**

To investigate the effects of participation in nursing student congresses and other motivation sources on students’ occupational motivation states.

**Methods:**

This descriptive pilot study covers 29 students who are studying in nursing department. The answers given to the “Motivation’s Resources and Problems Scale” (MRPS) were evaluated with Mann-Whitney U (MWU) test which is one of the non-parametric tests.

**Results:**

The mean age of participants was 19.2±0.9 years. There was no significant difference between the students who participated and those who did not participate in MRPS total scores. However, even though the difference was not significant, medical vocational high school students’ internal motivation levels were found to be higher than the other school graduates.

**Conclusions:**

There was no significant difference between the ranking of the scores that are acquired from motivation scale and participation status of nursing student in the congress. On the other hand, there are other factors that motivate occupational learning of students. Planning studies about increasing nursing students’ positive motivation sources is suggested.

## INTRODUCTION

Motivation is a decision-making process in which an individual acts on reaching a certain goal. It is the reason behind every action that a person performs to reach a destination.^1^ Studies show that the concept of motivation covers a wide range of motives named as needs, interests, goals and preferences.^2^

Motivation is individual actions that are performed which changes according to experiences, culture and needs.^3^,^4^ Motivation starts with individual’s discern on not yet actualized desires or needs, and in order to achieve these the individual takes action.^5^ The researchers classify motivation in two components i.e as internal and external. Internal motivation is considered as a natural learning source by educators. The studies reveal that the ones with higher internal motivation are more successful in realizing their goals than the others.^1^,^4^,^6^,^7^ For students, an education meeting the expectations, appreciating student’s knowledge and skills, and grading are among the external motivation sources.^4^,^7^,^8^

Professional motivation of a student is an important factor for competitiveness. Professional motivation is a system of goals and needs that motivates the student to acquire new knowledge and skills and to develop a conscious attitude towards the profession. Since a student with less than enough motivation would not be ready to learn, student motivation is one of the biggest challenges with which educators face.^8^

Educators can provide students’ learning behaviors by using different motivation methods. A student comes to the class with a certain motivation level. However, the behaviors of the educators, teaching style, class’ structure, task’s structure and social interactions have great influence on student motivation.^9^ In order to provide better motivation and performance, external motivation must be transformed to internal motivation. Since students can show reluctance, apathy and resistance while carrying out the action of learning with external motivation, internal motivation is preferred by educators because it leads a creative and high-quality learning and integrates one’s knowledge and beliefs with successful behaviors.^7^

Some of the external motivation support internal motivation. Satisfying a student’s sense of belonging to a community can be ensured by encouraging them to participate in group studies or social activities.^9^ Students may choose their education of a profession that is very different from the main profession they want to adopt as career in the future. Therefore, creating professional motivation requires more control, management and effort.^2^ It is possible to save the program from monotony by diversifying the teaching methods, offering learning options, giving them the opportunity to prepare successfully for tasks and presentations, inviting guests who are related with lectures or professions, cooperating with related organizations, individuals and peers.^10^

While the desire to help others and to do something useful is the main motivation for nursing students, it has been found that, mostly for male students, the nursing profession provides job security, opportunities and flexibility as well as the desire to give care to others.^11^

To be a good nurse, having an academic career or using knowledge for personal development may be the internal motivation of student nurses. Scientific and social activities are important academic organizations in terms of the sense of professional belonging, sense of usefulness to society and professional motivation. In order to create learning environments that support learning, the factors affecting the professional motivation of nursing students should be determined. This study was conducted with the aim of investigating the effects of participating in nursing student congresses and other motivation sources on students’ professional motivation situations.

## METHODS

This descriptive study consisted of twenty-nine (n=29) students 1st year students of the Nursing Department of the School of Health Sciences of Toros University who accepted to participate. Twelve students who participated in the Nursing Students Congress in Eskisehir on 2016 and 17 students who did not participate in the Congress were asked to answer the questions in Motivation’s Resources and Problems Scale. Nursing Students Congress was organized in Osmangazi University Faculty of Health Sciences in Eskisehir, Turkey from April 28-29^th^, 2016. The main theme of the congress was “Nursing and Technology”.

The necessary permissions for the study was obtained. Informed verbal consents from the study participants was obtained by the researchers by explaining the aim of the study and confidentiality was assured. The data were collected at the end of the course with the permission of the related instructor. Face to face interviews with the students were arranged to collect data under the supervision of the researcher. It took about 10 minutes to fill in the data forms. After the questionnaires were distributed to the students willing to participate in the study, they were asked to read each statement carefully before responding. Of the 12 students participating in the congress, 66.6% were female and 33.4% were male. Of the 17 students who did not participate, 76.4% were female and 23.6% were male. The mean age of the students who participated in the congress was X=19.5 and the mean age of the ones who did not participate was X=19.3, and the youngest one was 19 years old and the oldest one was 21.

### Motivation Resources and Problems Scale (MRPS)

Motivational Resources and Problems Scale (MRPS), which consisted of 24 questions by Acat and Kosgeroglu was used. The internal consistency (Cronbach’s alpha coefficient) of the scale prepared by Acat and Kosgeroglu (2006) was reported as 0.82.^12^ The reliability of the scale was sufficient when the Cronbach’s alpha criterion was taken lowest as 0.7 which is reported by Nunnaly (1978).^13^

The scale has three sub-dimensions. The internal motivation subscale consists of 11 items, the negative motivation subscale has eight items and the external motivation subscale consists of five items. The items numbered 1, 2, 3, 4, 6, 7, 8, 9, 10, 23, 24 are used for obtaining internal motivation scores, items numbered 5, 11, 12, 16, 18, 19, 21, 22 are used for obtaining negative motivation scores and items numbered 13, 14, 15, 17, 20 are used obtaining external motivation scores.

### Rating of the scale

In the items that constitute the internal and external motivation subscales, the answer ’I completely disagree’ is 1, ‘I disagree’ is 2, ’I’m undecided’ is 3, ’I agree’ is 4 and ’I absolutely agree’ is 5 points. In the items that constitute the negative motivation subscale, the ’I completely disagree’ is 5, ’I disagree’ is 4, ’I’m undecided’ is 3, ’I agree’ is 2 and ’I absolutely Agree’ is 1 points. The score of each subscale is determined by taking the arithmetic mean of the scores obtained from the items of the subscale. The average of the scores of the three subscales obtained is the score of the Occupational Learning Motivation Level of the person. It is recommended that the scale be used in the collection of data to be made in nursing education programs and educational settings and in the studies on the motivation levels of the students receiving nursing education.^14^

### Data Analysis

The data obtained from the survey were analyzed using SPSS 20 package program. For data analysis, arithmetic mean, standard deviation and percentage ratios were calculated. And in the statistical evaluation, p<0.05 was considered significant.^15^ The answers given to the “Motivation’s Resources and Problems Scale” were evaluated with Mann-Whitney U (MWU) test which is one of the non-parametric tests. MWU: Known as small sample test. N<20 is preferred in the studies. This test was used because the number of students attending the congress was 12 and the number of students who did not attend was 17.

## RESULTS

Of the students participating in the study, 79.3% were female and 20.7% were male. The mean age was 19.2±0.9 years (F:19.1±0.8 years, M:19.7±1.2 years). The Mann-Whitney U test was used to determine whether there was a significant difference between the students’ scores on MRPS and Motivation Resources subscales and their participation in Nursing Student Congress and some of their characteristics.

The findings obtained from the Mann Whitney-U test revealed that there was no statistically significant difference between the total scores of MRPS of the students who did or did not participate in the congress and their participation statuses to Nursing Student Congress (U: 84.500). It was also found that there was no significant difference between the total scores got from the MRPS and their gender, the school they graduated and their reasons for choosing the department. The MWU test results are as follows, respectively: (U: 47.500), (U: 70.000) and (U: 98.000). Table-I.

**Table-I:** Scores of motivation and motivation subscales according to some characteristics of students.

Characteristics		Scale Total Scores		Internal motivation		External motivation		Negative motivation	

	n	Average	Total	Average	Total	Average	Total	Average	Total
*Participation*									
Yes	12	16.46	197.50	14.92	179.00	17.13	205.50	16.42	197.00
No	17	13.97	237.50	15.06	256.00	13.50	229.50	14.00	238.00
		U:84.500 Z:0.779, p:0.436		U:101.000 Z:0.044, p:0.965		U:79.500 Z:-1.159, p:0.247		U:85.000 Z:-0.755, p:0.450	
*Gender*									
Female	23	15.93	366.5	15.85	364.5	15.50	356.5	15.85	364.5
Male	3	11.42	68.5	11.75	70.5	13.08	78.5	11.75	70.5
		U:47.500 Z:-1.163, p:0.254		U:49.500 Z:-1.053, p:0.302		U:57.500 Z:-0.635, p:0.546		U:49.500 Z:-0.635, p:0.302	
*Type of graduation school*									
Vocational	19	16.32	310.0	16.39	311.50	15.87	301.50	16.18	307.50
Other	10	12.50	125.00	12.3	123.50	13.35	133.50	12.75	127.50
		U:70.000 Z:-1.153, p:0.249		U:68.500 Z:-1.219, p:0.223		U:78.500 Z:-0.777, p:0.437		U:72.500 Z:-1.035, p:0.301	
*Department preference status*									
Willingly	12	14.67	176.00	14.79	177.50	14.42	173.00	15.13	181.50
Un-Willingly	17	15.24	259.00	15.15	257.50	15.41	262.00	14.91	253.50
		U:98.000 Z:-0.178, p:0.859		U:99.500 Z:-0.111, p:0.912		U:95.000 Z:-0.318, p:0.750		U:100.50 Z:-0.067, p:0.947	

P: 0.05

In our study, whether there is a significant difference between the scores of the “Internal Motivation Resources” subscale, “External Motivation Resources” subscale and the “Negative Motivation Resources” subscale in MRPS and the participation to the congress was again evaluated with the Mann Whitney-U Test. According to this; there was no significant difference between the scores obtained from the “Internal Motivation Resources” subscale and participation in the Nursing Student Congress (U: 101.000). There was no significant statistical difference between the scores of the “External Motivation Sources” (U: 79.500) and the “Negative Motivation Resources” subscales (U: 85.000) and their participation in the congress.

There was no significant difference between the scores of the students that they got from “Internal Motivation Sources”, “External Motivation Resources” and “Negative Motivation Resources” subscales in MRPS and their gender, the school that they graduated and their reasons for choosing the department. However, even though the difference is not significant, the students who graduated from medical vocational high school have higher internal motivation levels than the other school graduates. The MWU test results for the sub-scales are given below. “Internal Motivation Resources” subscale results respectively are; (U: 49.500), (U: 68.500) and (U: 99.500). “External Motivation Resources” subscale results respectively are; (U: 57.500), (U: 78.500) and (U: 95.000). “Negative Motivation Resources” subscale results respectively are; (U: 49.500), (U: 72.500) and (U: 100.50).

## DISCUSSION

Internal motivation for a nursing student can be listed as being a good nurse, an academic career or wanting to use the health information for his own development. The studies have revealed that the ones with higher internal motivation are more successful than the others.^7^,^16^ External motivation sources can be listed as teaching satisfaction, willingness of the group to be educated, appreciation of the students during their correct use of their knowledge and skills, etc. The fact that an individual considers themself inefficient, does not try to learn, is under pressure, or makes mistakes causes negative motivation.^12^

In our study, internal motivation levels of the students participating in the congress were found 14.92, external motivation levels were found 17.13, negative motivation levels were found 16.42). The internal motivation levels of the students who did not participate in the congress were found 15.06, the external motivation levels were found 13.50 and the negative motivation levels were found 14.00. Considering the lowest and highest scores that can be obtained from the scale, it is seen that the internal motivation scores are low, and the external and negative motivation scores are at the medium levels.

In the studies by Celik et al., it was observed that the internal and external motivation levels of the student nurses were high and the negative motivation levels were low.^7^ In the study performed by Gencay and Gencay, it was determined that the students’ external motivation average was higher.^17^ In their study, Karaman et al. found that the negative motivation score of the student nurses was higher than the other types of motivation.^8^

Although there are not enough studies in our country regarding the occupational motivation of nursing students, it has been observed that the interest in the occupations in the health sector has increased recently due to economic reasons. In Koksal and Yurttas’s (2015) studies, it was determined that 57% of the students preferred nursing profession due to economic reasons.^18^ This situation is thought to be effective on students’ higher external motivation score.^19^

There is no statistical difference between the genders of the students and their internal and external motivation levels. Similar to this finding of our study, result of the study conducted by Dundar et al. (2007), also showed that there was no significant difference in internal and external motivation according to gender.^20^ This is an important finding. Because the process of getting male students into nursing schools in our country is very new. The fact that men have similar occupational motivations with women can be interpreted as they embraced the nursing profession as women.

There is no statistical difference between motivation scores and the fact that students’ department choices are positive or negative. In other words, the students are motivated at the same level regardless of the conditions they prefer. It is thought that the increase in the popularity of nursing profession will be an effective factor in the emergence of this situation. As a matter of fact, in the studies of Nazik et al. (2014), it was determined that the students saw the profession of nursing among the ideal professions.^21^ Korkmaz and Ipekci (2015) found that more than half of the students placed nursing as the first choice.^22^

In our country, students who graduate from both vocational and regular high schools are accepted in nursing undergraduate programs. The fact that nursing students are educated at high school level is a topic that has been discussed for years and the literature supports the nursing education to be at the undergraduate level.^23^,^24^ Medical vocational high school students’ internal motivation levels are higher than the other school graduates. However, this difference was not statistically significant. The lack of a significant difference between the occupational motivations of the students and the schools they graduated, suggests that the vocational high school graduates cannot create enough awareness about their occupations and they cannot reveal a difference in the occupational motivation.

According to the regions where students live, there is no statistical difference between the occupational motivation scores. The acceptance of the nursing profession by the society is considered to be an effective factor in such outcome.

### Limitations of the Study

This study was conducted only on nursing students studying at one university. The sample size is also very small. Another limitation of the study was the fact that not all variables that could affect the level of occupational motivation in students were examined. For these reasons, the results of the study are valid only at Toros University and do not reflect the factors affecting the occupational motivation of other nursing students.

## CONCLUSION

The participation of the students in the nursing student congress who participated in the study did not affect the level of occupational motivation. However, such events are very useful in the formation of professional consciousness. It was observed that participating in a student congress for the first time and only as a listener was not enough to create a high level of excitement and motivation in students. In order to ensure adequate motivation, students may be encouraged to be supported to participate in the congress in order to present an event or presentation. We recommended to carry out further research on similar and other factors affecting occupational motivation.

### Author’s Contribution: 

All the authors contributed to the design of the study, collection of data, analysis, interpretation of data, and writing and approval of the study.
